# Sexual violence, deviance, and the paraphilias in American psychiatry, 1952–2013

**DOI:** 10.1080/09612025.2023.2197792

**Published:** 2023-05-03

**Authors:** Joanna Bourke

**Affiliations:** Department of History, Classics, and Archaeology, Birkbeck, University of London, London, England

**Keywords:** Sexual violence, rape, psychiatry, paraphilia, diagnostic statistical manual (DSM), sexual sadism, paedophilia, paraphilic coercive disorder, violent predator

## Abstract

This article explores arguments within American psychiatry from the 1950s around whether rapists were mentally ill. It analyses debates in the lead-up to the various editions of the American Psychiatric Association’s (APA’s) *Diagnostic and Statistical Manual of Mental Disorders* (DSM) from 1952 to the latest version in 2013, focussing particularly on a diagnostic category called ‘Paraphilic Coercive Disorder’ (PCD). Since the first DSM, American psychiatrists had routinely considered people who committed certain forms of sexual violence to be suffering from mental disorders. For example, ‘Paedophilia’ and ‘Sexual Sadism’ had always been considered valid diagnoses. However, the APA *refused* to pathologise non-sadistic sexual violence committed against adults. Opposition can be classified into four overlapping arguments: uncertainty about ‘normal’ male sexual aggression, feminist worries about ‘excusing’ harmful sexual behaviours, concerns about the misuse of psychiatry in courts, and the need to defend the psychiatric profession from encroachments on their ‘territory’ by non-medically trained psychologists, social workers, and anti-psychiatric activists.

This article explores arguments within American psychiatry from the 1950s around whether rapists who were neither paedophiles nor sadists were mentally ill. Work Groups responsible for adjudicating on which sexual disorders should be included in the *Diagnostic and Statistical Manual of Mental Disorders* (DSM), the so-called ‘Bible’ of the American Psychiatric Association (APA), have debated this question in every edition of the DSM, from 1952 to the latest version in 2013. The most heated discussions took place around proposals to introduce a diagnostic category called ‘Paraphilic Coercive Disorder’ (PCD). The term draws on the concept of ‘Paraphilias’, from ‘para’ or deviation and ‘filia’ meaning attraction. PCD, therefore, is defined as a pathological state that impelled sufferers (assumed male) to engage in sexually aggressive practices. Its inclusion in the DSM would classify rapists as mentally ill. In the lead up to the publication of DSM-III-R in 1987, arguments were particularly fraught, although the APA’s Board of Trustees eventually rejected the inclusion of PCD by a vote of ten to four.^1^

These debates are important because decisions about what diagnoses to include or exclude in the DSM could have momentous effects. Receiving a DSM-approved diagnosis affects treatment options, provides access to insurance and service provisions, defines eligibility to state benefits, and is a powerful force in determining levels of stigmatisation as well as sympathy. Not only is the DSM manual the obligatory authority on psychiatric diagnoses in North America, but it is also widely consulted in many other countries. It plays a central role in decisions made about diagnostic categories adopted in the *International Classification of Disease* (the ICD), which is used in over 140 member countries of the World Health Organization. This means that when the various Work Groups debated whether or not PCD, ‘Paraphilic Rapism’, or similar diagnostic labels should be included in the manual, they were aware of its potential to radically affect the lives of millions of people, as well as influence decisions made by physicians, insurers, social workers, lawyers, and penal officials.

Of course, debates about whether rapists were mentally ill long predated the publication of the first DSM in 1952.^2^ Since the late nineteenth century, alienists, psychiatrists, psychologists, and other medical professionals had frequently addressed the question. The most influential nineteenth and early twentieth century commentators were Iwan Bloch, Jean-Martin Charcot, Paul-Emile Garnier, Richard Von Krafft-Ebing, Albert Moll, and Ambroise Tardieu. Their arguments have been explored by numerous historians, most notably Ivan Crozier and Alison Moore, but also by me in *Rape: A History from the 1869s to the Present*.^3^

Much less attention has been paid to twentieth and early twenty-first century debates about the pathological nature of sexual violence. A notable exception is Jemma Tosh’s *Perverse Psychology. The Pathologization of Sexual Violence and Transgenderism* (2014).^4^ Although Tosh’s book explores some of the questions that will be addressed in this article, we differ about the medicalisation of rapists. Tosh admits that the psychiatric profession decisively rejected PCD, but then insisted that, nevertheless, the ‘remedicalisation of rape threatened to expand psychiatry’s jurisdiction by developing a diagnostic concept that would have been much broader than the “sexual sadism” diagnosis, a diagnosis that is often conflated with BDSM communities’.^5^ In contrast, I will be arguing, the APA consistently *refused* to pathologise non-sadistic sexual violence committed against adults. The elite of the psychiatric profession maintained a distinction between arousal to pain (pathological sadism) and arousal to non-consent (criminal rape). Tosh also insufficiently distinguishes between very different disciplines involved the debates around PCD, an error particularly important when she conflates psychology with psychiatry. I agree with Tosh about the ‘pathologization of transgenderism’ and the conflation of non-consensual sadism with consensual S&M practices within American psychiatry are convincing, which is why transgenderism is not explored in this article.

This article addresses arguments about the ‘medicalisation’ or ‘pathologisation’ of everyday life from the 1970s onwards.^6^ Medicalisation involves the practice of incorporating an expanding range of behaviours or identities into a medical paradigm. Numerous scholars have criticised the DSM for the way it has facilitated the rapid encroachment of psychiatric classifications into everyday life.^7^ One indication of the process can be inferred from the enlargement of the DSM itself. The first manual in 1952 was only 132 pages long; by 1980, it had grown to nearly 500 pages; and to 886 by 1994. The latest edition consists of 947 pages. Psychologists Roland Gori and Marie-José Del Volgo have even coined the term ‘pathologization of existence’.^8^ Implicit in such arguments is *over*-medicalisation as a form of labelling and social control. Medicalisation is conceived of as a way of dealing with and managing social dangers and marginalisations. The paradigmatic example of this is the medicalisation of deviance, a topic that has generated a very sophisticated historiography.^9^ The most influential is Thomas Szasz’s 1961 classic, *The Myth of Mental Illness*, which argued that mental disorders are not scientific entities, but value judgments imbued with strong political meanings and with the goal of enforcing social norms.^10^ A well-known example is homosexuality. It was only removed from the manual of psychiatric disorders in 1973, after members of the APA voted by a majority of 58% to accept the decision of the APA’s Board of Trustees to remove homosexuality from the DSM.^11^ As we shall see, while homosexuality was a rare example of a clinical diagnosis that moved *out* of the DSM, PCD is a diagnosis that sought, and failed, to be *included* in the manual. This article argues that the proposal to pathologise rapists led to impassioned debates within psychiatry because of difficulties distinguishing between ‘normal’ and ‘abnormal’ male sexual aggression. Arguments about introducing this new psychiatric diagnosis into the DSM were also inextricably tied to feminist analyses about the nature of rape and inadequacies of legal responses, concerns about the relationship between psychiatry and law, and attempts by a beleaguered psychiatric profession to defend its claims to scientific expertise.

## The DSM and the pathologisation of ‘deviant sexuality’

In order to understand the heated arguments about PCD, ‘Paraphilic Rapism’, and similar diagnostic terms such as ‘Sexual Assault Disorder’, it is necessary to observe that, since the first DSM, American psychiatrists had routinely, and without controversy, considered people who committed certain forms of sexual violence to be suffering from mental disorders. For example, ‘Paedophilia’ and ‘Sexual Sadism’ have always been considered valid diagnoses. In the first two editions of the DSM in 1952 and 1968, both terms appeared under the broad category of ‘Sexual Deviations’. The diagnoses were ‘reserved for deviant sexuality’ that were ‘not symptomatic of more extensive syndromes, such as schizophrenic and obsessional reactions’. They sat alongside other ‘deviations’ which were not sexually abusive, such as homosexuality, ‘transvestitism’ [*sic*], and fetishism.^12^ From DSM-III (1980), the ‘Sexual Deviations’ category was renamed ‘Paraphilias’. Psychiatrists consulting the manual were informed that the ‘essential feature’ of paraphilias was ‘unusual or bizarre imagery or acts’ being ‘necessary for sexual excitement’.^13^ Crucially, as we shall see, DSM-III added a residual category called ‘Atypical Paraphilia’ (later editions called it ‘Paraphilia NOS’ or ‘Paraphilia Not Otherwise Specified’), a ‘catch-all diagnosis’ or ‘diagnosis of convenience’ that was to create problems.

Only seven years after DSM-III was published, psychiatric thinking in the APA had changed so significantly that a ‘revised’ edition was believed to be required. DSM-III-R (1987) purported to be more ‘scientific’, in the sense that all diagnoses had to be empirically validated by medical research. The editors wanted to ensure that there were reduced opportunities for clinical subjectivity and they claimed (falsely) that the new diagnostic categories were not dependent on any specific theoretical position, by which they primarily meant psychoanalysis. The editors contended that a quantitative, biological-centred approach was more useful not only to physicians but also to insurance and pharmaceutical companies.^14^ Each paraphilia was described in terms of behaviours (A criteria) and distress or impairment (B criteria). For example, the A criteria for ‘Sexual Sadism’ was.

Over a period of at least 6 months, recurrent, intense sexually arousing fantasies, sexual urges, or behaviors involving acts (real not simulated) in which the psychological or physical suffering (including humiliation) of the victim is sexually exciting to the person.

The ‘B’ criteria maintained that ‘the fantasies, sexual urges, *or* behaviors cause clinically significant distress or impairment in social, occupational, or other important areas of function’.^15^ The ‘or’ rather than ‘and’ in the B criteria was later claimed to have been an editorial error. The disorder was never intended to be diagnosed on the grounds of behaviours alone. This error was to become significant during debates about diagnosing PCD.

The inclusion of sexually abusive practices such as ‘Paedophilia’ and ‘Sexual Sadism’ in every edition of the DSM served as models for psychiatrists lobbying for the addition of *other* forms of sexual violence in the manual. In 1976, for example, the first draft of what would become DSM-III (1980) included the diagnosis ‘Sexual Assault Disorder’. The A criteria was ‘the fantasy of sexual assault is erotically exciting’. The B criteria was.

There is significant motivation to translate the exciting fantasy into action. The individual has committed an act of sexual assault, or inevitably will in the near future. If the act has been committed in the past, there is significant motivation to repeat it.^16^

This classification was eventually rejected.

However, this debate marked the beginning of a period of concerted attempts in some psychiatric circles to pathologise non-sadistic rape of adults. In 1977, for example, two particularly influential arguments for the inclusion were made in the *American Journal of Psychiatry* and the *American Journal of Orthopsychiatry*. The authors were A. Nicholas Groth (psychologist at the Centre for Diagnosis and Treatment of Sexually Dangerous Persons in Bridgewater, Massachusetts), psychiatric nurse practitioner Ann Wolbert Burgess, and feminist sociologist Lynda Lytle Holmstrom. Three years earlier, Burgess and Holmstrom had carried out the first detailed research into the ‘normal’ responses of women who had experienced sexual assault. They coined the term ‘Rape Trauma Syndrome’.^17^ In their work with Groth, however, they focused on examining men who *inflicted* such harms. In their article in the *American Journal of Psychiatry*, the three authors concluded that none of the rapists in their study had expressed sexual motivations: they were driven by anger and the need to assert power.^18^ In ‘Rape: A Sexual Deviation’, published in the *American Journal of Orthopsychiatry*, Groth and Burgess went even further. They acknowledged that there were major differences between ‘sexual deviations’ and ‘sexual offences’. After all, not every psychiatrically ‘abnormal’ sexual behaviour was illegal (for example, masochism was not a crime); conversely, not every illegal behaviour was ‘abnormal’ (at the time, fellatio, even within marriage, was prohibited in many U.S. states). They asked: what makes a specific sexual activity a form of mental disorder? Their definition of sexual deviance was a conservative one. It included any ‘sexual behaviour in the service of non-sexual needs’. In other words, any sexual act that was ‘not primarily or essentially sexual in nature’ and which ‘jeopardize[d] the physical or psychological safety of another’ was psychiatrically deviant behaviour. Based on this definition, they appealed to the committee drafting the new edition of DSM-III to consider including rape as a paraphilia, since it was ‘a pseudosexual act in which the primary motive is not one of physical gratification’.^19^ Their arguments were drawing on the insights of second-wave feminists, most notably, Susan Brownmiller’s 1975 classic *Against Our Will*, where she argued that rape was an act of power, not sex. Translated into the language of Groth, Burgess, and Holmstrom, power and domination rather than sexual arousal were ‘non-sexual needs’.^20^ This meant that rapists warranted their own diagnostic label in the DSM.

Many other psychiatrists, psychologists, and sexologists in this period agreed. For example, in 1984, the distinguished (albeit controversial) sexologist John Money lobbied for the inclusion of diagnostic categories such as ‘biastrophilic rapism’ (‘βῐ́ᾱ’ is Greek for violence) or ‘raptophilia’ (‘rapto’ is Latin for ‘seized’ or raped) into psychiatric nomenclatures.^21^ He defined paraphilic rape as a syndrome in which the terror of the victim was an integral part of the ‘assailant’s lovemap’.^22^ Psychiatrist Gene G. Abel and psychologist Joanne-Lucine Rouleau made similar arguments in 1990. They contended that because ‘many individuals report having recurrent, repetitive, and compulsive urges and fantasies to commit rape’, it should be classified as a paraphilia.^23^ In the discussions leading to the 2013 publication of DSM-5, advocacy for the inclusion of PCD was particularly animated. The drafters of that edition proposed two diagnostic criteria for PCD:

(i) over a period of at least six months, recurrent, intense sexually arousing fantasies or sexual urges focused on sexual coercion; (ii) the person is distressed or impaired by these attractions, or has sought sexual stimulation from forcing sex on three or more non-consenting persons on separate occasions.^24^

They also maintained that if the ‘patient’ met the criteria for ‘Sexual Sadism’, that diagnosis should be made instead of PCD.

## The failure to pathologise rapists

Why did these attempts to pathologise rapists fail? Opposition can be classified into four overlapping arguments: uncertainty about ‘normal’ male sexual aggression, feminist worries about ‘excusing’ harmful sexual behaviours, concerns about the misuse of psychiatry in courts, and the need to defend the psychiatric profession from encroachments on their ‘territory’ by non-medically trained psychologists, social workers, and anti-psychiatric activists.

From the late 1970s, psychiatrists, psychologists, and social workers became increasingly interested in ‘normal’ male sexual behaviours and desires, unlike their predecessors who had focused primarily on ‘abnormal’ behaviours. In a series of in-depth interviews, questionnaires, and laboratory experiments, they found that a significant proportion of men were sexually aroused by images and descriptions of sexual coercion. For example, in 1980, when Claude Crépault and Marcel Couture interviewed ninety four fairly ordinary American men about their sexual fantasies, they discovered that 30% sometimes fantasised about raping women.^25^ Later that decade, a study involving American male college undergraduates revealed that over one third agreed with the statement that ‘I fantasize about raping a woman’. Two thirds responded positively to statements such as ‘I get excited when a woman struggles over sex’, ‘It would be exciting to use force to subdue a woman’, and ‘I fantasize about having a woman tied up, spread-eagled to a bed’.^26^ Even more disturbing, psychologist Neil M. Malamuth’s 1981 study exposed the fact that over one-third of male college students admitted that they would rape a woman if they were guaranteed not to be caught. Crucially, in contrast to men who reported relatively low sexual arousal to rape depictions and low acceptance of rape myths, men who had reported high levels on both scales resembled convicted rapists. The high scoring men were also more likely to respond with aggression against women in laboratory settings. Malamuth argued that this suggested that there was a link between *fantasies* of non-consent and actual *behaviour*.^27^ Such research led clinical social worker Jerome C. Wakefield to propose in 2011 that ‘the capacity for arousal under coercive conditions’ could be seen as a ‘normal part of sexuality that is usually suppressed by most individuals but can emerge under some conditions’.^28^ It was a dispiriting vision of the sexuality of American men.

If it was true that ‘normal’ male sexuality included ‘recurrent, intense sexually arousing fantasies or sexual urges focused on sexual coercion’ (the proposed diagnostic criteria for PCD in DSM-5) and if there was a strong relationship between a young man’s theoretical ‘propensity to rape’ and him engaging in sexually aggressive practices (that is, seeking ‘sexual stimulation from forcing sex’), then the proposed PCD diagnosis could apply to up to one-third of American men. This anxiety was augmented by the realisation that the research had been conducted on middle class, usually university educated, young American men. The equally privileged professionals conducting the surveys worried that introducing PCD into the DSM might generate a large number of ‘false positives’, leading to the pathologisation of young, middle-class, and predominantly white men. Arguments against including PCD in the diagnostic manual were a way to *defend* male privilege.

Feminist opponents of the proposed PCD diagnosis were not concerned about risks to so-called ‘normal’ male aggressors. But they were troubled that the diagnosis would be used to *excuse* sexual abusers and rapists. This was the second opposition. Paula Joan Caplan, who had served as an adviser to the DSM panel contended that if ‘Paraphilic Rapism’ was introduced into the DSM ‘a rapist’s tendency to think a lot about rape would be used in court by defence lawyers to argue that rapists should not go to prison but instead into psychiatric treatment’. To those who argued that her worries were unwarranted, Caplan responded that.

It was clear that the diagnosis would be used in this way because that was already happening even though the category was not yet listed in the DSM; therapists hired by the defense would interview rapists and testify that the fellows couldn’t help themselves and, in essence, weren’t evil but just emotionally disturbed and therefore ought not to go to jail but needed therapy.^29^

Caplan was equally appalled when the editors of DSM-IV-TR (eventually published in 2000) proposed to introduce ‘Masochistic Personality Disorder’ alongside ‘Paraphilic Rapism’. Caplan complained that this would mean that rapists were being excused for crimes on the grounds that they were mentally ill while victims of abuse could be dismissed as ‘masochists’ and thus partly responsible for the violence men inflicted upon them.^30^

Other feminists shared Caplan’s concerns.^31^ In an article published in the journal *Women Against Violence*, social worker Samantha Clavant registered her dismay that the proposed introduction of the diagnosis PCD would medicalise male violence and contribute to lessening the ‘responsibility for sexual assault from perpetrators’. She argued that feminism scholarship had shown that sexual abuse was a political issue, emerging within societal contexts that were damaging to girls and women. Clavant warned that perpetrators of sexual violence would become ‘victims of a disorder’. Even worse, men who committed *multiple* offences would be considered ‘sicker’ and ‘therefore have a stronger argument for mitigating circumstances in sentencing’. It would be relatively easy for men’s rights activists and defence teams to ‘utilise this to their advantage’, she contended.^32^

## Sexual violence predator laws

These feminist concerns were an important strand in the arguments against PCD. But even more vociferous opposition focused on connections between the proposed diagnoses and Sexually Violent Predator (SVP) laws that began to be introduced in most U.S. states from 1990.^33^ These laws were the unintended consequence of the introduction of fixed prison sentences, which coincided with a moral panic in the 1980s about a ‘wave’ of sexual violence, particularly against children. Prior to the 1980s, prison sentences imposed for sexual crimes were indeterminate: judges could exercise discretion in the severity of punishments meted out to violent offenders. With the rise of civil and human rights movements, attention was drawn to the biases (particularly racial ones) of judges. The introduction of *fixed* sentences for specific crimes was intended to serve the needs of justice. However, it also meant that judges had little discretion when sentencing men they believed were dangerous offenders and who had a history of recidivism or who were considered to be highly likely to reoffend. For example, the penalty for rape had been set at seven years, which had been calculated by simply averaging the range of sentences that had previously been given.^34^ This policy of averaging sentences had been adopted in order not to vastly increase the number of prison cells that would be required. Many commentators in the legal and justice professions believed that, when dealing with such offenders, the new fixed guidelines were releasing some of the most dangerous criminals from prison earlier than they would have been under the older system.

Public alarm peaked in 1990. The ‘trigger’ was the case of Earl Kenneth Shriner, a white offender in Washington State who had been convicted of the assault, rape, and attempted murder of a seven-year-old boy. He had originally been sentenced to 131 years’ imprisonment. However, changes in sentencing meant that he was released early and, almost immediately, he committed a brutal attack on two young girls. The state legislature of Washington responded by passing a SVP law. Within a short period, other states followed their example. These laws ruled that criminals who had completed their prison sentence for sexually violent crimes could be sent to an inpatient psychiatric institution indefinitely if they, first, were deemed likely to reoffend and, second, were diagnosed with a mental disorder. This meant that psychiatrists, psychologists, and other medical professionals were asked by the courts to adjudicate not only on the risk of a person reoffending, but also on the presence of a mental disorder. They turned to the DSM in order to diagnose an accredited mental illness. The most common diagnosis was ‘Pedophilia’,^35^ followed by ‘Paraphilia NOS’ and ‘Sexual Sadism’.

This wasn’t the first time that psychiatrists had been asked to be actively involved in the incarceration of sexually deviant criminals. The most important precedent occurred in the mid-1930s when many U.S. states introduced sexual psychopath laws to deal with persistent or violent offenders. However, there was one crucial difference. Under the *earlier* sexual psychopathic laws, which varied state by state, it was usual for an offender to be given a hearing in front of a jury *prior to conviction*. At the hearings, psychiatrists were asked to testify as to whether or not the offender suffered from a ‘criminal sexual psychopathic perversion’. If so, he could be committed to the psychiatric wing of the prison and, once psychiatrists within the hospital judged the offender to have been ‘cured’, he could be either placed on probation and released or returned to court and sentenced for the original crime. In contrast, under SVP laws, offenders served their prison sentences *after which* they were committed to a secure hospital. This was an important change for two reasons. First, it exposed a profound sense of disillusionment with psychiatry as a *curative* science. Psychiatric researchers had found that treatment regimes had little or no effect on the propensity of rapists and other sex offenders to reoffend.^36^ If violent men could not be cured through psychiatric interventions, then the rationale for releasing them after a period of treatment fell away. Second, commitment under SVP statutes effectively meant imprisonment for a very long time, even for life.

The laws shocked many psychiatrists, not least because of concerns about ‘double jeopardy’ (that is, once a person has been acquitted, convicted, and/or punished, they cannot be prosecuted again for the same crime) and ‘due process’ (or the rights of an accused person to be treated fairly and equally according to established legal procedures and rules).^37^ But, they were also dismayed that the laws threatened to fold ‘criminal behavior’ into ‘mental illness’.^38^ How were psychiatrists to ascertain whether or not a particular offender was unable to control himself? Since the link between ‘volitional impairment and sexual dangerousness’ had not been clinically confirmed, warned Richard Wollbert and Allen Frances (who had been Chair of DSM-IV Task Force), how were psychiatrists to ‘reliably differentiate SVPs from more typical recidivists who may be sexually dangerous but lack a mental abnormality’?^39^ Frances complained that legal documents typically characterised ‘predators’ as people who possessed ‘congenital or acquired conditions’ that affected their emotional or volitional capacities in such a way that ‘predisposed’ them to commit sexually violent offenses.^40^ But such a definition was ‘so broad’ that it ‘really provides no guidance’.^41^ Indeed, as Michael B. First (one of the editors of DSM-IV-TR) and psychiatrist Robert L. Hanlon contended, the ‘line between an irresistible impulse and an impulse not resisted is probably no sharper than that between twilight and dusk’.^42^

## The status of psychiatry as a ‘Science’

But there were other problems. In most cases, the people authorised to carry out the evaluations were not medically-trained psychiatrists, but psychologists and social workers. Law courts preferred such evaluators: they were much cheaper to employ than psychiatrists.^43^ They also had extensive experience working within justice or prison systems, so had very different interests to psychiatrists. Because the editors of the DSM had consistently rejected including PCD or ‘Paraphilic Rapism’ in the manual, these evaluators were relying on diagnoses such as ‘Pedophilia’, ‘Sexual Sadism’, and ‘Paraphilia, NOS’. Their interpretation of these diagnoses focused on *behaviours* (that is, they had committed an offence), despite the fact that DSM criteria stipulated that patients also had to experience ‘fantasies and sexual urges’ that caused ‘clinically significant distress or impairment in social, occupational, or other important areas of function’.

Florida psychologist Gregory DeClue and Dennis Doren, a state hospital psychologist from Wisconsin, justified their reliance on behaviours. DeClue argued that ‘Paraphilia NOS (nonconsent)’ was a legitimate diagnosis if a person ‘repeatedly engages in sexual behaviors with nonconsenting persons over a period of at least six months’.^44^ For DeClue, it was obvious that.

It is not mentally normal to enjoy sexual behavior with nonconsenting persons. It is not mentally normal to prefer sexual behavior with nonconsenting persons and it is not mentally normal to be indifferent to whether one’s sexual partner/victim consents to sexual behavior or not.^45^

DeClue was ignoring or unaware of the work of academic psychologists mentioned earlier, which demonstrated that one-third of ‘normal’ American men *were* sexually aroused by non-consent.

Doren took such arguments further. His 2002 textbook entitled *Evaluating Sex Offenders: A Manual for Civil Commitments and Beyond* was extremely influential since the hostility of both the APA and the American Psychological Association to SVP laws meant that they refused to provide guidance on how to conduct evaluations. Doren’s manual became the ‘bible for many forensic evaluators in SVP commitment cases, especially those who testify primarily for the prosecution’.^46^ Doren acknowledged that diagnosing ‘Paraphilia NOS’ for the purposes of SVP assessments relied primarily on observed behaviours rather than fantasies and sexual urges. However, he contended, this was inevitable since convicted rapists nearing the end of their prison sentences would obviously be reluctant to admit in civil commitment proceedings that they were plagued by deviant fantasies and urges. After all, acknowledging such urges could result in them being incarcerated in a mental hospital for the rest of their lives. Doren used this argument to justify his contention that evaluators could make a diagnosis based on behaviour alone.^47^ In this way, ‘Paraphilia NOS’ effectively became a ‘proxy for rape’: if a person had committed the crime of rape, this alone was sufficient to diagnose them as suffering from a ‘Paraphilia NOS’.^48^

Prominent psychiatrists vehemently contested such arguments. First, they argued that it was wrong to diagnose a mental disorder from criminal behaviours alone. Simply because a man committed several rapes over a particular period did not mean that he met the criteria for a paraphilia, argued Michael B. First and Robert L. Hanlon. Even the fact that he ejaculated during the violence was not proof that he was ‘aroused by aggression’.^49^ After examining the case reports of nearly one hundred state psychological evaluators, Frances agreed. He observed that.

Their reports gave remarkably detailed descriptions of the offender’s criminal behavior, but provide little or no rationale or justification for the diagnosis of paraphilia. The write-ups are all long and thorough – but completely off the point and generic. … . The most common error was to assume that a behavior alone (the act of rape) can by itself qualify someone for a mental disorder diagnosis of Paraphilia.^50^

In a jointly authored article, Frances and First asked whether evaluators were aware of

rape for gain (e.g. by pimps or sex traffickers), opportunistic rapes, date rape, gang rape, rape for dominance, rape under the disinhibiting influence of substances, rape related to an antisocial personality pattern of criminality, and rape influenced by other mental disorders (e.g. mania or mental retardation)?^51^

Indeed, researchers discovered, convicted rapists resembled other *criminals* more than they resembled other *paraphilics*. They were the ‘most heterogeneous of sexual offenders’, observed Devon Polaschek. She concluded that the difficulties psychiatrists faced when diagnosing any of the paraphilias were more pronounced when diagnosing ‘rapism’, making any inclusion of PCD into the DSM ‘premature’.^52^

Second, psychiatrists contended that too many of the people lobbying for the inclusion of PCD or ‘Paraphilic Rapism’ in the DSM had a conflict of interest. They tended to be psychologists, social workers, and medical professionals working within carceral or semi-carceral institutions. This included psychiatrists such as Groth, who, as we have heard, was employed at the Centre for Diagnosis and Treatment of Sexually Dangerous Persons, which held men committed under psychopathic and SVP laws. There was outrage when, in the lead-up to the publication of DSM-5 in 2013, it was revealed that the Sub-Workgroup deciding on the paraphilias were ‘field testing’ the diagnosis in SVP commitment centres.^53^ Indeed, the two reports submitted to the Sub-Workgroup that supported the inclusion of PCD had been written by a Treatment Director of an SVP commitment centre and an SVP prosecutor.^54^

Third, they contended that SVP evaluators were misusing both the ‘Paraphilia NOS’ and the ‘Sexual Sadism’ diagnoses. The second most common diagnosis used in SVP proceedings was that ‘catch-all’ diagnosis ‘Paraphilias NOS’.^55^ But it did not even have set criteria. The DSM listed examples of ‘Paraphilias NOS’: they were telephone scatologia (obscene telephone calls), necrophilia (sexual intercourse with corpses), partialism (sexual interest in specific bodily parts such as feet, hair, and buttocks), zoophilia (sex with nonhuman animals), coprophilia (sexual arousal to faeces), klismaphilia (sexual enjoyment of enemas), and urophilia (sexual pleasure in urination)—none of which resembled criminal rape.^56^ Indeed, the different editions of the DSM explicitly stated that the ‘Not Otherwise Specified’ paraphilias were ‘less frequently encountered’, which was hardly a characterisation of rape.^57^ Yet, ‘Paraphilias NOS’ began appearing with great regularity in SVP proceedings. It was a ‘made-up diagnosis’, contended Frances, used in order to ‘justify the psychiatric commitment of rapists who without this “diagnosis” would be regarded as no more than common, if particularly heinous, criminals’.^58^

Faced with these criticisms over the use of ‘Paraphilia NOS’, SVP evaluators switched to the diagnosis of ‘Sexual Sadism’. However, this too was an extremely rare disorder. DSM-III claimed that ‘studies of rapists indicate that fewer than 10% have sexual sadism’.^59^ Most estimates were much lower. Numerous studies of convicted sex offenders or patients attending clinics specialising in paraphilias found less than 6% of the offenders/patients to be suffering from ‘Sexual Sadism’.^60^ Most of those diagnosed were serial murderers. In one analysis of nearly half a billion visits to U.S. outpatient medical clinics, there were *no* cases reported.^61^ Diagnosing ‘Sexual Sadism’ was nothing more than a ‘fad diagnosis’, claimed Frances and Wollert.^62^

What distinguished ‘Sexual Sadism’ from other forms of sexual violence? The various DSM committees expended considerable intellectual energy on this question. For them, the distinction rested on the offenders’ arousal patterns. For example, the editors of DSM-III-R (1987) contended that it was the ‘visible pain of the victim’, as opposed to non-consent *per se*, that sadists found ‘sexually arousing’. ‘Sexual Sadists’ were ‘*motivated* by the prospect of inflicting suffering’.^63^ They used ‘stereotyped and ritualistic violence’ to fulfil ‘deep-seated fantasies that are the main event of the sex act’, Frances explained.^64^ The distinction between arousal to *pain* compared to arousal to *non-consent* was important given the research mentioned earlier in this article about the high proportion of men who acknowledged being aroused by non-consent in masturbatory fantasies or who admitted that they would rape a woman if they could be assured of not being punished. In other words, leading members of the APA were engaged in a process of defining the parameters for what was ‘normal’ male sexual aggression. So long as it was not acted upon—or, at least, not in what was (very problematically) called an ‘excessive’ way—arousal to non-consent could be considered psychiatrically ‘normal’. If the violence was ‘excessive’, it was simply ‘criminal’.

## Professional gatekeeping

There was more at stake than simply the status of psychiatry as a precise science. The psychiatrists who argued against introducing PCD were also protecting their professional reputation. The anti-psychiatry movement had been growing increasingly vocal since Szasz’s *The Myth of Mental Illness* had been published in 1961. In the 1970s, the status of the APA had been bruised by campaigns by the gay community to remove homosexuality from the manual. By the 1980s, it was widely reported that American psychiatry was ‘undergoing an identity crisis’.^65^ It was accused of lacking transparency, engaging in social control, medicalising everyday behaviours, exploiting therapeutic relationships, harming distressed patients, and espousing racist and sexist views.^66^

The involvement of psychiatrists in SVP procedures threatened further loss of status. After all, SVP committals were very different to standard psychiatric commitment procedures. While most patients who were subjected to civil commitments were suffering from severe psychotic disorders,^67^ and were typically held for only short periods, men committed under SVP laws often showed no serious symptoms or distress yet their incarceration in mental institutions could last decades. In addition, as Frances complained, ‘the primary goal of SVP proceedings (however it is veiled) is to protect society, not the “patient”’.^68^ It was a classic example of the political misuse of psychiatry.^69^

The APA agreed. In 1999, they sided with the majority of their members in resisting calls to participate in SVP procedures. The APA claimed that this decision was due to their need to retain their integrity, which meant avoiding becoming ‘a tool of state oppression’.^70^ The APA’s Task Force on ‘Dangerous Sex Offenders’, which reported in 1999, contended that SVP statutes were an ‘unacceptable misuse of psychiatry’. The laws represented ‘a serious assault on the integrity of psychiatry’ and ‘threaten[ed] to undermine the legitimacy of the medical model of commitment’. The Task Force called on psychiatrists to ‘vigorously oppose these statutes in order to preserve the moral authority of the profession and to ensure continuing societal confidence in the medical model of civil commitment’.^71^

Leading psychiatrists lent their voice to these critiques, agreeing not only that participating in SVP proceedings was damaging, but that including diagnoses such as PCD in the DSM manual would undermine the status of psychiatry by conflating crime and mental disorders. For example, Abraham Halpern (President of The American Academy of Psychiatry and the Law) argued against introducing ‘Sexual Assault Disorder’ in DSM-III and PCD in DSM-5 on the grounds that it would ‘be an invitation to the anti-psychiatric movement to scorn and ridicule the American Psychiatric Association’.^72^ His concerns were echoed by psychiatrist and ethicist John Z. Sadler. For Sadler, introducing ‘vice-laden diagnoses’ would ‘perpetuate the public perception that psychiatrists are social control agents and that we serve as morality police, not physicians’.^73^ In effect, influential psychiatrists sided with ‘anti-psychiatry’ spokespeople in an attempt to reposition psychiatry as a caring profession.

## Conclusion

The various editors of the DSM considered, but ultimately rejected, including diagnoses such as PCD and ‘Paraphilic Rapism’ in their manual. This did not mean that the APA entirely gave up any plans to expand their scientific authority to encompass non-sadistic rapists of adults. Psychiatrists continued to treat patients struggling with aggressive urges. Indeed, DSM-IV and DSM-IV-TR introduced ‘V-codes’ precisely to deal with the ‘fuzzy boundaries’ of psychiatric diagnoses. These codes could be applied to patients whose problems might be ‘a focus of clinical attention’ even though they were not suffering from recognised mental disorders. V61.12, for example, was reserved for perpetrators who had abused their partners and V62.83 for those who abused non-partners.^74^ The editors of the most recent DSM opened the door to PCD being included in a future manual by mentioning the diagnosis in Section II (or the Appendix), which is where diagnostic categories that warrant further study are placed.

Although there were powerful voices on both sides of the debate, the victors were those who argued *against* the medicalisation of all forms of sexual violence, except ‘Pedophilia’, ‘Sexual Sadism’, and ‘Zoophilia’. This is why the historiographical focus on ‘medicalisation’ is misplaced in the context of sexual violence in the modern period, although it is a powerful concept for understanding nineteenth century psychiatric interventions, as argued by myself, Crozier, Moore, and Tosh, in particular. The refusal of elite psychiatrists to expand the reach of psychiatry was partly scientific—specifically, difficulties in distinguishing ‘criminal’ from ‘mentally disordered’ rapists. But they were also acting in the self-interest of their profession. Public opinion was also against the diagnosis. As Gene G. Abel and Joanne-L. Rouleau put it, ‘the scientific evidence must be balanced with society’s acceptance of such a categorization’.^75^ In coming to these decisions against the medicalisation of non-sadistic rape of adult women, the psychiatric authorities were engaged in complex negotiations with feminists, civil rights groups, and anti-psychiatry commentators, who had been emboldened by their success in getting homosexuality removed from the manual. Ironically, agreeing to limit their authority while affirming classificatory boundaries between criminal conduct and mental disorder served the medicalisation paradigm well by insisting upon the psychiatric profession’s scientific credentials. The decision also pointed to an underlying unease about ‘normal’ male sexuality and its discontents.

## Notes

1 Herb Kutchins and Stuart A. Kirk, *Making Us Crazy. DSM: The Psychiatric Bible and the Creation of Mental Disorders* (New York: Free Press, 1997).

2 American Psychiatric Association, *Diagnostic and Statistical Manual of Mental Disorders* (1st ed.) (Washington, DC: American Psychiatric Association, 1952).

3 Joanna Bourke, *Rape: A History from the 1860s to the Present* (London: Virago, 2007); Ivan Crozier, ‘James Kieran and the Responsible Pervert’, *International Journal of Law and Psychiatry* 25, no. 4 (July 2002): 331–50; Alison Moore, ‘The Invention of Sadism? The Limits of Neologisms in the History of Sexuality’, *Sexualities* 12, no. 4 (October 2009): 489–506; Alison Moore, ‘Rethinking Gendered Perversion and Degeneration in Visions of Sadism and Masochism, 1886–1930’, *Journal of the History of Sexuality* 18, no. 1 (January 2009): 138–57; Alison Moore, *Sexual Myths of Modernity: Sadism, Masochism and Historical Teleology* (Lanham: Lexington Books, 2015); Jemma Tosh, *Perverse Psychology. The Pathologization of Sexual Violence and Transgenderism* (London: Routledge, 2014).

4 Tosh, Perverse Psychology.

5 Ibid., 52.

6 Many commentators use medicalisation and pathologisation interchangeably. It is more accurate to use pathologisation when some kind of (somatic/psychological) event or behaviour is reframed as a disease or disorder or is seen as ‘abnormal’ while medicalisation is when medical professionals intervene to treat it.

7 This is a huge literature, but see Jennifer S. Blumenthal-Barby, ‘Psychiatry’s New Manual (DSM-5): Ethical and Conceptual Dimensions’, *Journal of Medical Ethics* 40, no. 8 (August 2014): 531–36; Allan V. Horwitz, *Creating Mental Illness* (Chicago: University of Chicago Press, 2002); Annemarie Goldstein Jutel, *Putting a Name to It: Diagnosis in Contemporary Society* (Baltimore: Johns Hopkins University Press, 2011); Martyn D. Pickersgill, ‘Debating DSM-5: Diagnosis and the Sociology of Critique’, *Journal of Medical Ethics* 40, no. 8 (August 2014): 521–25; Owen Whooley and Allan V. Horwitz, ‘The Paradox of Professional Success: Grand Ambition, Furious Resistance, and the Derailment of the DSM-5 Revision Process’, in *Making the DSM-5*, eds. Joel Paris and James Phillips (New York: Springer, 2013), 75–92.

8 Roland Gori and Marie-José Del Volgo, *La santé totalitaire: essai sue la médicalisation de l’existence* (Paris: Flammarion, 2005).

9 For example, see Kristin Barker and Tasha R. Galardi, ‘Diagnostic Domain Defense: Autism Spectrum Disorder and the DSM-5’, *Social Problems* 62, no. 1 (February 2015): 120–40; Peter Conrad, ‘Medicalization and Social Control’, *Annual Review of Sociology* 18 (1992): 209–32; Peter Conrad. The Shifting Engines of Medicalization’, *Journal of Health and Social Behavior* 46 (2005): 3–14; Peter Conrad and Joseph W. Schneider, *Deviance and Medicalization: From Badness to Sickness*, 1st 1980 (Philadelphia: Temple University Press, 1992); Irving Kenneth Zola, ‘Medicine as an Institution of Social Control’, *Sociological Review* 20 (1972): 487–504.

10 Thomas Szasz, *The Myth of Mental Illness: Foundations of a Theory of Personal Conduct* (New York: Paul B. Hoeber, 1961). Also see Kutchins and Kirk, *Making Us Crazy. DSM*; R. D. Laing, *The Politics of Experience* (London: Penguin Books, 1967); Thomas J. Scheff, *Being Mentally Ill: A Sociological Theory* (Chicago: Adine, 1966); Thomas J. Scheff, *Labeling Madness* (Englewood Cliffs: Prentice-Hall, 1975); Thomas Szasz, ‘The Myth of Mental Illness’, *American Psychologist* 15 (1960): 113–18.

11 Ronald Bayer, *Homosexuality and American Psychiatry: The Politics of Diagnosis* (Princeton: Princeton University Press, 1987); Rachel Bingham and Natalie Banner, ‘The Definition of Mental Disorder: Evolving but Dysfunctional?’ *Journal of Medical Ethics* 40, no. 8 (August 2014): 537–42; Ian Hacking, *Mad Travellers: Reflections on the Reality of Transient Mental Illnesses* (Charlottesville: University Press of Virginia, 1998).

12 American Psychiatric Association, *Diagnostic and Statistical Manual, Mental Disorders* (Washington DC: American Psychiatric Association, 1952), 38–9; American Psychiatric Association, *DSM-II. Diagnostic and Statistical Manual of Mental Disorders.* 2nd ed. (Washington DC: American Psychiatric Association, 1968), 10.

13 American Psychiatric Association, *Diagnostic and Statistical Manual of Mental Disorders.* 3rd ed.*. DSM-III* (Washington DC: American Psychiatric Association, 1980), 266.

14 Michael Strand, ‘Where Do Classifications Come From? The DSM-III, The Transformation of American Psychiatry, and the Problem of Origins in the Sociology of Knowledge’, *Theory and Society* 0.3 (May 2011): 273–313; Mitchell Wilson, ‘DSM-III and the Transformation of American Psychiatry: A History’, *American Journal of Psychiatry* 150, no. 3 (March 1993): 399–410.

15 American Psychiatric Association, *Diagnostic and Statistical Manual of Mental Disorders.* 4th ed.*. DSM-IV* (Washington DC: American Psychiatric Association, 1994), 530. Emphasis added.

16 Abraham Halpern, cited in ‘The Rejection of Paraphilic Rape: A First Hand Historical Narration’, *Psychiatric Times* (28 February 2011), np.

17 Ann Wolbert Burgess and Lynda Lytle Holmstrom, ‘Rape Trauma Syndrome’, *American Journal of Psychiatry* 981 (1974).

18 A. Nicholas Groth, Ann Wolbert Burgess, and Lynda Lytle Holmstrom, ‘Rape: Power, Anger, and Sexuality’, *American Journal of Psychiatry* 34 (1977): 1239–43.

19 A. Nicholas Groth and Ann Wolbert Burgess, ‘Rape: A Sexual Deviation’, *American Journal of Orthopsychiatry* 47, no. 3 (1977): 400.

20 Thomas K. Zander, ‘Commentary: Inventing Diagnosis for Civil Commitment of Rapists’, *Journal of the American Academy of Psychiatry and Law* 36 (2008): 460.

21 John Money, ‘Paraphilias: Phenomenology and Classification’, *American Journal of Psychotherapy* 38, no. 2 (April 1984).

22 John Money, *Lovemaps. Clinical Concepts of Sexual/Erotic Health and Pathology, Paraphilia, and Gender Transposition in Childhood, Adolescence, and Maturity*, 1st 1986 (New York: Irvington Publishers, 1993), 54.

23 Gene G. Abel and Joanne-L. Rouleau, ‘The Nature and Extent of Sexual Assault’, in *Handbook of Sexual Assault: Issues, Theories, and Treatment of the Offender*, eds. William Lamont Marshall, D. L. Laws, and Howard E. Barbaree (New York: Plenum Press, 1990), 18.

24 Anthony R. Beech and Leigh Harkins, ‘DSM-IV Paraphilias: Descriptions, Demographics, and Treatment Interventions’, *Aggression and Violent Behavior* 17 (2012): 532.

25 Claude Crépault and Marcel Couture, ‘Men’s Erotic Fantasies’, *Archives of Social Behavior* 9 (1980): 565–82.

26 Virginia Greendlinger and Donn Byrne, ‘Coercive Sexual Fantasies of College Men as Predictors of Self-Reported Likelihood to Rape and Overt Sexual Aggression’, *The Journal of Sex Research* 23, no. 1 (February 1987): 5.

27 Neil M. Malamuth, ‘Rape Proclivity Among Males’, *Journal of Social Issues* 37, no. 4 (1981): 138–57. There are serious problems in the design of this research. For this article, though, what matters is that the research was positively reviewed and widely accepted, not the scientific validity of their findings.

28 Jerome C. Wakefield, ‘DSM-5 Proposed Diagnostic Criteria for Sexual Paraphilias: Tensions Between Diagnostic Validity and Forensic Utility’, *International Journal of Law and Psychiatry* 34, no. 3 (2011): 207.

29 Paula Joan Caplan, *They Say You’re Crazy: How the World’s Most Powerful Psychiatrists Decide Who’s Normal* (Reading, M.A.: Addison-Wesley, 1995), 86.

30 Caplan, *They Say You’re Crazy*, 86–7.

31 These legal concerns were not only expressed by feminists: see Abraham Halpern, cited in ‘The Rejection of Paraphilic Rape’, np.

32 Samantha Clavant, ‘Concerns About the New Diagnostic and Statistical Manual of Mental Disorders’, *Women Against Violence: An Australian Feminist Journal* 22 (2010): 64–5.

33 These were sometimes called Sexually Dangerous Predator laws.

34 Allen J. Frances, ‘Sexually Violent Predator Statutes: The Clinical/Legal Interface’, *Psychiatric Times* (December 1, 2008), at https://www.psychiatrictimes.com/view/sexually-violent-predator-statutes-clinicallegal-interface.

35 Judith V. Becker and others, ‘Characteristics of Individuals Petitioned for Civil Commitment’, *International Journal of Offender Therapy and Comparative Criminology* 47 (2003): 185–95; W. Lawrence Fitch and Debra A. Hammen, ‘The New Generation of Sex Offender Commitment Laws: Which States Have Them and How do They Work?’ in *Protecting Society from Sexually Dangerous Offenders*, eds. Bruce J. Winick and John Q. La Fond (Washington DC: American Psychological Association, 2003), 27–59; Jill S. Levenson, ‘Sexual Predator Civil Commitment: A Comparison of Selected and Released Offenders’, *International Journal of Offender Therapy and Comparative Criminology* 48, no. 6 (2004): 638–48.

36 Janice K. Marques and others, ‘Effects of a Relapse Prevention Program on Sexual Recidivism: Final Results from California’s Sex Offender Treatment and Evaluation Program (SOTEP)’, *Sexual Abuse: A Journal of Research and Treatment* 17, no. 1 (January 2005), 79–107; Friedrich Lösel and Martin Schumucker, ‘The Effectiveness of Treatment of Sexual Offenders: A Comprehensive Meta-Analysis’, *Journal of Experimental Criminology* 1 (2005), 117–46; R. Karl Hanson, Ian Broom, and Marylee Stephenson, ‘Evaluating Community Sex Offender Treatment Program: A 12-Year Follow-up of 724 Offenders’, *Canadian Journal of Behavioural Science/Revue canadienne des sciences du comportment* 36, no. 2 (April 2004): 87–96.

37 Allen J. Frances, ‘Most Rapists are Not Sadists: How to Tell the Difference and Why it is So Important’, *Psychiatric Times*, 2 May 2011, at https://www.psychiatrictimes.com/view/most-rapists-are-not-sadists-how-tell-difference-and-why-it-so-important.

38 Howard Zonana and others, *Dangerous Sex Offenders: A Task Force Report of the American Psychiatric Association* (Washington DC: APA, 1999), 173–74.

39 Frances and Richard Wollert, ‘Sexual Sadism: Avoiding its Misuse in Sexually Violence Predator Evaluations’, *The Journal of the American Academy of Psychiatry and Law* 40, no. 3 (2012): 409.

40 Frances, ‘Sexually Violent Predator Statutes’.

41 Ibid.

42 Michael B. First and Robert L. Hanlon, cited in Robert A. Prentky, Anna I. Coward, and Adeena M. Gabriel, ‘Commentary: Muddy Diagnostic Waters in the SVP Courtroom’, *Journal of the American Academy of Psychiatry and Law* 36 (2008): 457.

43 Zander, ‘Commentary: Inventing Diagnosis for Civil Commitment of Rapists’, 459.

44 Gregory DeClue, ‘Paraphilia NOS (Nonconsenting) and Antisocial Personality Disorder’, *The Journal of Psychiatry and Law* 34, no. 4 (winter 2006): 508.

45 DeClue, ‘Paraphilia NOS (Nonconsenting) and Antisocial Personality Disorder’, 508.

46 Zander, ‘Commentary: Inventing Diagnosis for Civil Commitment of Rapists’, 460.

47 Dennis Doren, *Evaluating Sex Offenders: A Manual for Civil Commitments and Beyond* (Thousand Oaks: Sage Publications, 2002).

48 Christopher M. King and others, ‘Disputed Paraphilia Diagnoses and Legal Decision Making: A Case Law Survey of Paraphilia NOS, Nonconsent’, *Psychology, Public Policy, and Law* 20, no. 3 (2014): 295–96.

49 Michael B. First and Robert L. Hanlon, ‘Use of DSM Paraphilic Diagnoses in Sexually Violent Predation Commitment Cases’, *Journal of the American Academy of Psychiatry and Law* 36 (2008): 446.

50 Allen J. Frances, ‘Going for Wins in Sexually Violent Predator Cases’, *Psychiatric Times*, 8 June 2011, at https://www.psychiatrictimes.com/view/going-wins-sexually-violent-predator-cases. Emphasis added. Also see Allen J. Frances, ‘California DMH Instructs SVP Evaluators on Proper DSM-IV-TR Diagnosis’, 5 January 2012, at https://www.psychiatrictimes.com/view/california-dmh-instructs-svp-evaluators-proper-dsm-iv-tr-diagnosis; Allen J. Frances, ‘Another Step Towards Ending’, 10 October 2011, np.; Allen J. Frances, ‘My Review of 28 Sexually Violent Predator Cases’, *Psychology Today*, 29 March 2012, at https://www.psychologytoday.com/intl/blog/dsm5-in-distress/201203/my-review-28-sexually-violent-predator-cases.

51 Allen Frances and Michael B. First, ‘Paraphilia: NOS, Nonconsent: Not Ready for the Courtroom’, *Journal of the American Academy of Psychiatry and Law* 39 (2011): 559. Also see Frances, ‘My Review of 28 Sexually Violent Predator Cases’.

52 Devon L. L. Polaschek, ‘The Classification of Sex Offenders’, in Tony Ward, D. Richard Laws, and Stephen M. Hudson (eds.), *Sexual Deviance. Issues and Controversies* (Thousand Oaks: Sage Publications, 2003), 161.

53 Andrew Hinderliter’s commentary in James Philips and others, ‘The 6 Most Essential Questions in Psychiatry Diagnosis: A Pluralogue Part 2: Issues of Conservatism and Pragmatism in Psychiatric Diagnosis’, *Philosophy, Ethics, and Humanities in Medicine* 7 (2012): 5.

54 Andrew Hinderliter’s commentary in Philips and others ‘The 6 Most Essential Questions in Psychiatry Diagnosis’, 5.

55 The most common was ‘Pedophilia’.

56 APA, *DSM-IV*, 532.

57 Ibid., 523.

58 Allen J. Frances, ‘Rape, Psychiatry, and Constitutional Rights – Hard Cases Make for Very Bad Law’, Psychiatric Times, 1 September 2010, at https://www.psychiatrictimes.com/view/rape-psychiatry-and-constitutional-rightshard-cases-make-very-bad-law. Also see Allen Frances, Shoba Sreenivasan, and Linda E. Weinberg, ‘Defining Mental Disorder When it Really Counts: DSM-IV-TR and SVP/SDP Statutes’, *Journal of the American Academy of Psychiatry and Law* 36 (2008): 381; Allen J. Frances, ‘DSM5 and Sexual Disorders – Just Say No’, *Psychiatric Times*, 18 March 2010, at https://www.psychiatrictimes.com/view/dsm5-and-sexual-disorders--just-say-no; Frances, ‘The Latest in Sexually Violent Predator’, 30 January 2015, np; Frances and First, ‘Paraphilia: NOS, Nonconsent’, 555–61; Allen J. Frances, ‘Going for Wins in Sexually Violent Predator Cases’, *Psychiatric Times*, 8 June 2011, at https://www.psychiatrictimes.com/view/going-wins-sexually-violent-predator-cases.

59 APA, *DSM 3rd edition Revised*, 287–88. Emphasis added.

60 Able and Rouleau, ‘The Nature and Extent of Sexual Assault’, 9–21; Stephen H. Allnutt and others, ‘Co-Morbidity of Alcoholism and the Paraphilias’, *Journal of Forensic Science* 41, no. 2 (1996): 234–39; Reinhard Eher and others, ‘Stable Dynamic Risk Factors in Child Sexual Abusers: The Incremental Predictive Power of Narcissistic Personality Traits Beyond the Static-99/Stable-2007 Priority Categories on Sexual Reoffense’, *Sexual Offender Treatment* 5, no. 1 (2010): 4; Richard B. Krueger, ‘The DSM Diagnostic Criteria for Sexual Sadism’, *Archives of Sexual Behavior* 39 (2010): 325; Zander, ‘Commentary: Inventing Diagnosis for Civil Commitment of Rapists’, 460; Howard Zonana and others, *Dangerous Sex Offenders: A Task Force Report of the American Psychiatric Association* (Washington DC: APA, 1999).

61 Frances and Wollert, ‘Sexual Sadism’, 409.

62 Ibid., 414.

63 APA, *DSM 3rd Edition Revised*, 287–88. Emphasis added. Also see Abel and Rouleau, ‘The Nature and Extent of Sexual Assault’, 19.

64 Allen J. Frances, ‘Most Rapists are Not Sadists: How to Tell the Difference and Why it is So Important’, *Psychiatric Times*, 2 May 2011, at https://www.psychiatrictimes.com/view/most-rapists-are-not-sadists-how-tell-difference-and-why-it-so-important.

65 Michał Wróblewski, ‘The DSM as a Moving Laboratory: The Role of the Diagnostic Manual in the Stabilizing and Objectification of Pharmaceutical Reason’, *Polish Sociological Review* 189 (2015): l93.

66 See Bourke, ‘Historical Perspectives on Mental Health and Psychiatry’, in *Mind, State, and Society. Social History of Psychiatry and Mental Health in Britain, 1960–2010*, eds. George Ikkos and Nick Bouras (Cambridge: Cambridge University Press, 2021), 3–12, at https://www.cambridge.org/core/books/mind-state-and-society/historical-perspectives-on-mental-health-and-psychiatry/93E5F3EE5E829C40528B74D0379EB9D1

67 Thomas K. Zander, ‘Civil Commitment Without Psychosis: The Law’s Reliance on the Weakest Links in Psychodiagnosis’, *Journal of Sexual Offender Civil Commitment* 1 (2005): 17–82.

68 Allen J. Frances, ‘Sexually Violent Predator Statutes: The Clinical/Legal Interface’, *Psychiatric Times*, 1 December 2008, at https://www.psychiatrictimes.com/view/sexually-violent-predator-statutes-clinicallegal-interface.

69 Frances, ‘Most Rapists are not Sadists.

70 Frances, ‘Rape, Psychiatry, and Constitutional Rights’.

71 Howard Zonana and others, *Dangerous Sex Offenders: A Task Force Report of the American Psychiatric Association* (Washington DC: APA, 1999), 173–74.

72 Abraham Halpern, cited in ‘The Rejection of Paraphilic Rape’, np.

73 John Z. Sadler, ‘Vice Squad’, *Psychiatric Times*, 27 August 2010, at https://www.psychiatrictimes.com/view/vice-squad.

74 APA, DSM Fourth Edition. Text Revision DSM-IV-TR, 731 and 738.

75 Abel and Rouleau, ‘The Nature and Extent of Sexual Assault’, 189

